# The Lung Immune Prognostic Index Discriminates Survival Outcomes in Patients with Solid Tumors Treated with Immune Checkpoint Inhibitors

**DOI:** 10.3390/cancers11111713

**Published:** 2019-11-02

**Authors:** Daniel E. Meyers, Igor Stukalin, Isabelle A. Vallerand, Ryan T. Lewinson, Aleksi Suo, Michelle Dean, Scott North, Aliyah Pabani, Tina Cheng, Daniel Y.C. Heng, D. Gwyn Bebb, Don G. Morris

**Affiliations:** 1 Department of Oncology, University of Calgary, Calgary, AB T2N 4N2, Canada; daniel.meyers@ucalgary.ca (D.E.M.); istukali@ucalgary.ca (I.S.);; 2 Division of Dermatology, University of Calgary, Calgary, AB T2N 2T9, Canada; 3 Faculty of Kinesiology, University of Calgary, Calgary, AB T2N 1N4, Canada; 4 Cross Cancer Institute, Edmonton, AB T6G 1Z2, Canada

**Keywords:** immunotherapy, immune checkpoint inhibitors prognostic biomarkers, LIPI, NSCLC, RCC, melanoma, real-world evidence

## Abstract

Immune checkpoint inhibitors (ICI) have revolutionized the treatment landscape of several solid tumor types. However, as patient outcomes are heterogeneous, clinical tools to aid in prognostication are needed. The Lung Immune Prognostic Index (LIPI) correlates with outcomes in patients with non-small cell lung cancer (NSCLC) treated with ICI, but its applicability beyond NSCLC is poorly defined. We sought to determine whether LIPI is associated with overall survival (OS), progression-free survival (PFS) and objective response rate (ORR) in a pooled, real-world, retrospective cohort of patients with solid tumors treated with ICI. Of the total pooled cohort (*N* = 578), 47.2%, 38.2% and 14.5% of patients were stratified into good, intermediate and poor LIPI group, respectively. Median OS were 22.8 (95% CI 17.4–29.5), 7.8 (95% CI 6.6–9.6), and 2.5 months (95% CI 1.4–3.4) (*p* < 0.0001). Median PFS were 9.9 (95% CI 7.2–11.5), 3.6 (95% CI 2.7–4.3), and 1.4 months (95% CI 1.2–2.2) (*p* < 0.0001). ORR was also associated with LIPI group (*p* < 0.001). Intermediate and poor LIPI were independently prognostic of OS compared to good LIPI, with hazard ratios (HR) of 1.8 (95% CI 1.4–2.3, *p* < 0.001) and 3.6 (95% CI 2.5–5.1, *p* < 0.001), respectively. These data are the first to suggest that in a real-world setting, the prognostic value of LIPI may be tumor agnostic.

## 1. Introduction

The treatment of metastatic cancer has seen a paradigm shift over the last decade with the advent of immune checkpoint inhibitors (ICI). The efficacy of ICI has clearly been demonstrated in a number of phase III randomized control trials in non-small cell lung cancer (NSCLC) [1,2], renal cell carcinoma (RCC) [3,4], and melanoma [5]. Nevertheless, heterogeneity in outcomes remains with only 20–40% of patients having an objective response. Further, ICI are known to be associated with a host of immune-related adverse events [6], and carry a significant financial burden [7]. Therefore, identification of robust prognostic biomarkers are needed to aid in appropriate treatment allocation.

Although the body of literature exploring prognostic biomarkers of ICI outcomes is growing, a single ubiquitous clinical tool is lacking. Pre-treatment tumor expression of PD-L1 represents the most well-studied biomarker to date. Theoretically, tumor expression of the immunosuppressive PD-L1 protein suggests the presence of antitumor T-cells within the tumor microenvironment, and thus disruption of this axis would re-invigorate anti-tumor immunity [8]. More recently, tumor mutational burden (TMB) has been proposed as a biomarker of response to ICI, as higher prevalence of somatic mutations may expose infiltrating T-cells to neoantigens—ultimately stimulating an antitumor immune response. Despite the successful application in some cases [9,10], neither tumor PD-L1 or TMB are sufficient on their own [11]. Further, routine testing of these indices are not readily available in all centers, and may not be cost-effective. As such, there has been a recent surge in research efforts to determine how readily available clinical and serological parameters may inform prognostication in patients treated with ICI.

Notably, Mezquita and colleagues [12] have developed the Lung Immune Prognostic Index (LIPI)—a simple, readily translatable clinical tool that stratifies patients in “poor”, “intermediate” and “good” prognostic groups based on a pre-treatment lactate dehydrogenase (LDH) greater than the upper limit of normal (ULN) and a derived neutrophil-to-lymphocyte ratio (dNLR) ≥3. Other groups have further corroborated the value of LIPI in NSCLC, with a focus on clinical trial populations [13,14]. However, it has yet to be determined if LIPI has prognostic utility in a tumor agnostic fashion—especially in the real-world.

As such, we sought to investigate the role of LIPI in prognosticating clinical outcomes in a pooled, real-world, multi-center cohort of patients with solid tumors treated with ICI. As a secondary objective, we evaluated the prognostic value of LIPI in our individual NSCLC, RCC, and melanoma cohorts.

## 2. Results

### 2.1. Baseline Characteristics

In total, 643 patients were identified as having received ICI for NSCLC, RCC or melanoma. After exclusion of those patients with insufficient data to allocate a LIPI score within 1-month of ICI initiation, 578 (90.0%) patients were included in the final analysis (Table 1). The final pooled cohort consisted of patients with NSCLC (52.2%), RCC (25.1%) and melanoma (22.7%).

Of the pooled cohort, 47.2%, 38.2% and 14.5% of patients were stratified into good, intermediate and poor LIPI group, respectively. The median age at treatment initiation with ICI was 66.7 years, and 65.6% of patients were less than 70 years of age. Further, more patients had Eastern Cooperative Oncology Group performance status (ECOG PS) scores of 0/1 (73.5%) than 2/3 (25.1%) and were treated with ICI in the 2nd, or later, (74.6%) line of therapy more than 1st line (25.4%). At the time of analysis, 31.1% of patients were alive and had a median follow-up time of 23.5 months (range 1.8–89.0 months).

Baseline characteristics of the individual cohorts are summarized in Supplementary Table S1.

### 2.2. Overall Survival (OS)

**Figure 1:**
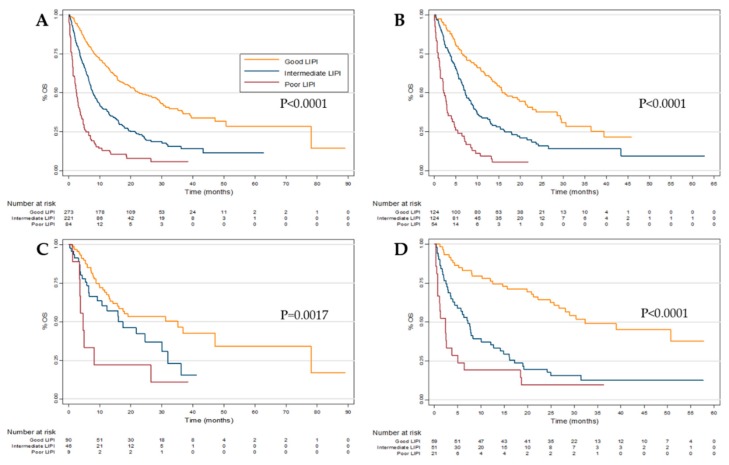
Overall survival (OS) according to Lung Immune Prognostic Index (LIPI) group in the (**A**) pooled, (**B**) Non-small cell lung cancer (NSCLC), (**C**) Renal cell carcinoma (RCC) and (**D**) melanoma cohorts.

The median OS from the initiation of any ICI in the pooled cohort was 10.9 months (95% CI 9.1–13.2 months). Furthermore, the median OS were 22.8 (95% CI 17.4–29.5), 7.8 (95% CI 6.6–9.6) and 2.5 (95% CI 1.4–3.4) months in the good, intermediate and poor LIPI groups, respectively (*p* < 0.0001) (Figure 1A). In a multivariate model accounting for patient age, tumor type, ECOG PS, and line of therapy, both intermediate (HR, 1.8; 95% CI 1.4–2.3, *p* < 0.001) and poor (HR, 3.6; 95% CI 2.5–5.1, *p* < 0.001) LIPI scores were still associated with worse OS. (Table 2)

Patient stratification by LIPI was also significantly associated with survival outcomes in the individual NSCLC, RCC, and melanoma cohorts (Figure 1B–D), and median OS values are specifically quantified in Supplementary Table S2. Using the same multivariate model as outlined above, both intermediate and poor LIPI groups were significantly (*p* < 0.005) associated with worse OS in both the the NSCLC and melanoma cohorts (Supplementary Tables S3 and S4**)**. In the RCC cohort only the poor (*p* < 0.005), but not intermediate (*p* = 0.25), LIPI group was significantly associated with survival (Supplementary Table S5).

### 2.3. Progression-Free Survival (PFS)

**Figure 2:**
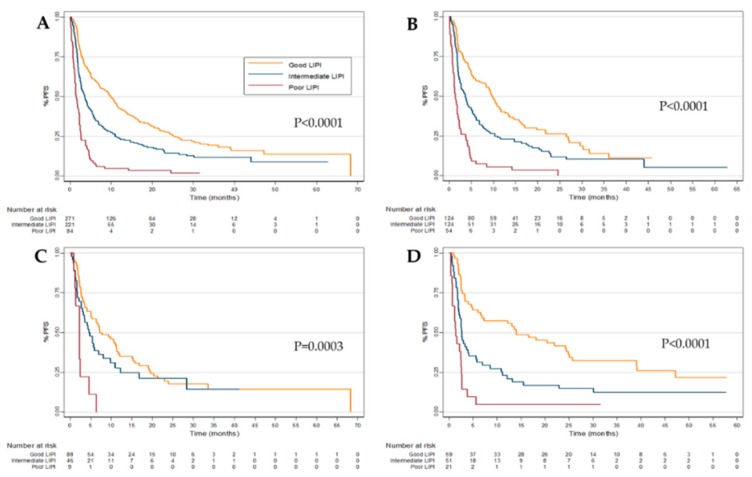
Progression-free survival (PFS) according to Lung Immune Prognostic Index (LIPI) group in the (**A**) pooled, (**B**) Non-small cell lung cancer (NSCLC), (**C**) Renal cell carcinoma (RCC) and (**D**) melanoma cohorts.

The median PFS for the combined cohort was 4.5 months (95% CI 3.7–5.3 months). PFS were 9.9 (95% CI 7.2–11.5), 3.6 (95% CI 2.7–4.3) and 1.4 (95% CI 1.1–2.2) months in the good, intermediate and poor LIPI groups, respectively (*p* < 0.0001) (Figure 2A). Using the same multivariate model as above, both intermediate (HR, 1.3; 95% CI 1.1–1.7, *p* = 0.02) and poor (HR, 3.0; 95% CI 2.0–4.5, *p* < 0.001) LIPI groups were still associated with worse PFS. (Table 2)

As is consistent with the OS data presented above, patient stratification by LIPI was also significantly associated with PFS in the individual NSCLC, RCC, and melanoma cohorts (Figure 2B–D, Supplementary Table S2). On multivariate analysis, both intermediate and poor LIPI were associated with worse OS in the NSCLC (*p* < 0.05) and melanoma (*p* < 0.001), but as with the OS data, this association was only seen in RCC within the poor LIPI group. (Supplementary Tables S3–S5).

### 2.4. Objective-Response Rate (ORR)

The ORR for the pooled cohort receiving ICI was 24.6%. Notably, 14.4% of patients either had significant clinical deterioration, or death, prior to receiving radiologic assessment after ICI initiation.

As summarized in Table 3, LIPI was significantly associated with ORR (*p* < 0.001). On multivariate analysis, LIPI was also a significant independent prognostic factor for ORR in the intermediate (OR, 1.7; 95% CI 1.1–2.6, *p* = 0.018) and poor (OR, 9.9; 95% CI 3.4–28.5, *p* < 0.001) groups. (Table 4) In the individual cohorts, only poor LIPI in NSCLC (*p* < 0.005) and melanoma (*p* < 0.005), and intermediate LIPI in melanoma (*p* < 0.001) were significantly associated with ORR. (Supplementary Table S6).

## 3. Discussion

The advent of ICI has dramatically altered the treatment landscape of a number of solid tumor types, and some patients with previously incurable diseases are now experiencing durable clinical benefit [15,16]. However, there is substantial clinical heterogeneity as the majority of patients treated with ICI still do not have an objective response to therapy [8]. As such, clinical strategies to aid in prognostication of treatment outcomes are needed.

A landmark study by Mezquita and colleagues demonstrated that a simple clinical tool—LIPI—using baseline LDH and dNLR had significant association with treatment outcomes in patients with NSCLC [12]. In this multi-centre, real-world retrospective cohort study, we sought to evaluate the utility of LIPI in discriminating between clinical outcomes in patients treated with ICI in a tumor agnostic fashion. Further, we sought to explore the prognostic value of LIPI in individual cohorts of patients with NSCLC, RCC, and melanoma.

In our pooled cohort of 578 patients, median OS and PFS from the initiation of ICI were 10.9 and 4.5 months, respectively. Patients in the poor LIPI group were significantly more likely to have no objective clinical response to ICI (OR, 9.9; *p* < 0.001), progress sooner on treatment (HR, 3.0; *p* < 0.001) and have worse survival outcomes (HR, 3.6; *p* < 0.001) compared to those in the good LIPI group, even when accounting for important clinical factors such as age, ECOG PS, and line of therapy. Further discrimination of outcomes was seen in the intermediate LIPI group, as they also had worse ORR (OR, 1.7; *p* = 0.018), PFS (HR, 1.3; *p* = 0.019) and OS (HR, 1.8; *p* < 0.001) compared to the good LIPI group. These data are the first to highlight the potential for LIPI to be integrated into the clinical arena beyond NSCLC. Of course, prospective validation of these findings is required.

Moreover, we also demonstrated that LIPI maintains prognostic value in individual cohorts of NSCLC, RCC, and melanoma. These data support previously published findings in NSCLC [12,13,14], but are the first to demonstrate an association between LIPI and meaningful clinical outcomes in both RCC, and melanoma. Interestingly, the discriminatory value of LIPI in RCC was limited to the poor group, as there was no difference in OS, PFS, or ORR between the good and intermediate LIPI groups.

Why might LIPI be effective in a tumor agnostic fashion? The relationship between the immune system, carcinogenesis, and disease progression has been more clearly elucidated in recent years and inflammation is now recognized as a “hallmark” of cancer [17]. As such, indicators of systemic inflammatory status should theoretically apply to all immunogenic malignancies, which in this case include NSCLC, RCC, and melanoma. Specifically, both LDH and dNLR have been identified as potential inflammatory biomarkers, and linked with poor clinical outcomes in cancer [18,19,20]. However, these readily collected serologic variables are not alone in this regard, which ultimately leaves room for the improvement of LIPI. Currently, available data also implicate albumin [21], the platelet-to-lymphocyte ratio [20], and even body mass index [22] as being potential markers of inflammatory status and specifically, associated with outcomes with ICI.

It is important to note that our study included a sizeable group of patients with ECOG PS ≥2, representing 25.1% of the dataset. As such, this would point towards the applicability of our data to patients who would’ve otherwise been excluded by most clinical trial protocols. Despite the absolute necessity of properly designed randomized clinical trials in advancing the therapeutics available for our patients, a current shortcoming is their stringency of inclusion and exclusion criteria. Ultimately, in the current age, populations studied in the clinical setting diverge from the patients encountered in real-world clinical practice. Herein lies the benefit of real-world data—they provide the opportunity to evaluate the applicability of clinical trial findings and may stimulate valuable clinical inquiry. For example, in our pooled cohort, ECOG PS ≥2 (HR, 2.3; *p* < 0.01) was significantly and independently associated with poor survival outcomes. This suggests that in addition to serologic data such as LDH and dNLR, clinical level data such as ECOG PS may also enhance prognostication in the ICI era.

Areas of future investigation should seek to prospectively validate our current findings. Furthermore, the LIPI score dichotomizes both dNLR and LDH in a binary fashion, even though they are continuous variables. As such, it remains to be seen whether the cut-off points for LIPI can be optimized. Finally, as machine learning approaches have proven useful in predicting clinical outcomes in medicine [23,24], an intriguing idea is the application of this approach to predict outcomes with ICI.

Limitations of this study are its retrospective nature, which led to the exclusion of 10% of identified patients due to missing data. Further, diagnostic imaging was reviewed locally, which may limit the strength of the ORR data.

## 4. Materials and Methods

### 4.1. Study Design

We conducted a multicenter retrospective cohort study at two tertiary cancer centers in Canada—the Tom Baker Cancer Centre in Calgary, Alberta and the Cross Cancer Institute in Edmonton, Alberta. Inclusion criteria for this study were: patient age >18 years, histologically confirmed NSCLC, RCC or melanoma, and receipt of ICI (nivolumab, pembrolizumab, ipilimumab/nivolumab) between 1 January 2010 and 1 June 2019. Patients were identified consecutively using provincial pharmacy records. The sole exclusion criteria was insufficient data to calculate pre-treatment LIPI, as outlined below. Data collection and chart review occurred between 1 July 2017 and 1 July 2019.

Baseline demographic, clinical, pathological and serological data were collected for each patient. We used a data cutoff of 30-days prior to initiation of ICI for serological data. Radiologic assessments were performed at the discretion of the attending physician, and responses were graded per RECIST (Response Evaluation Criteria in Solid Tumors) v1.1. Physician review of radiology reports was conducted if explicit recording of tumor response and/or progression was unclear.

The LIPI score was calculated as previously defined [12], using LDH > upper limit of normal and dNLR >3 as cutoff points. dNLR is calculated as follows: ((absolute neutrophil count)/ (total leukocyte count—absolute neutrophil count)). As such, patients could be assigned a score of 0, 1 or 2 based on their LDH and dNLR values, corresponding to good, intermediate and poor LIPI, respectively.

Access of patient records was approved by Health Research Ethics Board of Alberta—Cancer Committee (17-0125). Individual patient consent was not required due to the retrospective nature of this study.

### 4.2. Statistical Analysis

Patient characteristics were compared using chi-squared or Fisher’s exact tests. The primary endpoint of our study was overall survival (OS), which was calculated from the date of immunotherapy initiation until death of any cause, or last patient follow-up. Patients who did not die within the study period were right censored. These patients contributed person-time until their last follow-up. Our secondary outcomes included Progression-free survival (PFS) and Objective response rate (ORR). PFS was calculated from the date of immunotherapy initiation until earliest of radiologic progression, death from any cause, or time of last follow-up. ORR was defined as the proportion of patients who achieved a complete or partial response and patients with un-evaluable responses were considered as non-responders [25].

Survival analyses were performed using the Kaplan-Meier method and log-rank tests. Cox proportional hazards models were constructed for each cohort to identify individual factors associated with OS and PFS. Univariate and multivariate models for OS, PFS, and ORR associated with LIPI status were created [12], including baseline age (<70 vs. ≥70 years) cohort type (NSCLC, RCC or melanoma), line of therapy (1 vs. ≥2), and ECOG status (<2 vs. ≥2). Patients with missing data (*n* = 8) were excluded from multivariate analysis. Interactions between ECOG and LIPI status were assessed using Wald tests. A backward elimination method was used to construct models with all potentially relevant covariates and eliminate each covariate one at a time to observe the change on the estimated HRs. A change of >10% to the estimated HR suggested the presence of a confounding variable. Final models were determined based on adjustment for confounding variables. The proportional hazards assumption was assessed using Schoenfeld residuals and log-log plots where violations were detected [26,27]. In cases where the proportional hazards assumption was not met, models were truncated by excluding 20% of early events. A p-value of less than 0.05 was considered statistically significant for all analyses. Statistical analyses were performed with Stata v14.2 (College Station, Texas, USA).

## 5. Conclusions

Herein, we have demonstrated for the first time that LIPI can discriminate clinical outcomes, including ORR, PFS, and OS, amongst patients treated with ICI in a tumor agnostic fashion. Further, we have independently validated the utility of LIPI in NSCLC, as well as demonstrated its prognostic value in both RCC and melanoma. Importantly, these findings arise from a real-world data set, making them applicable to patients seen in every day clinical practice. Prospective validation of LIPI as a prognostic biomarker in the era of ICI is warranted.

## Figures and Tables

**Table 1:** Baseline clinical and demographic information of the pooled cohort.

Parameter	Pooled Cohort (*N* = 578)
**LIPI Group—n (%)**	
Good (0)	273 (47.2)
Intermediate (1)	221 (38.2)
Poor (2)	84 (14.5)
**Cohort—n (%)**	
NSCLC	302 (52.4)
RCC	145 (25.1)
Melanoma	131 (22.7)
**Age**	
Median (years)	66.7
Range (years)	32.5–87.2
<70—n (%)	379 (65.6)
≥70—n (%)	199 (34.4)
**Treatment Line—n (%)**	
1	147 (25.4)
≥2	431 (74.6)
Range	1–5
**ECOG PS—n (%)**	
<2	425 (73.5)
≥2	145 (25.1)
Unknown	8 (1.4)
**Alive at Analysis—n (%)**	180 (31.1)
Median follow-up (months)	23.5
Range (months)	1.8–89.0

ECOG PS: Eastern Cooperative Oncology Group performance status, LIPI: lung immune prognostic index.

**Table 2:** Multivariate analysis of factors associated with overall survival (OS) and progression-free survival (PFS) in the pooled cohort.

Parameter	OS		PFS	
	HR (95% CI)	*p* Value	HR (95% CI)	*p* Value
**LIPI Group**				
Good (0)	1.0 (reference)		1.0 (reference)	
Intermediate (1)	1.8 (1.4–2.3)	<0.001	1.3 (1.0–1.7)	0.019
Poor (2)	3.6 (2.5–5.1)	<0.001	3.0 (2.0–4.5)	<0.001
**Cohort**				
NSCLC	1.0 (reference)		1.0 (reference)	
RCC	0.6 (0.5–0.8)	0.002	1.1 (0.8–1.4)	0.65
Melanoma	0.8 (0.6–1.1)	0.18	0.9 (0.7–1.3)	0.68
**Age**				
<70	1.0 (reference)		1.0 (reference)	
≥70	0.9 (0.7–1.2)	0.60	1.0 (0.8–1.2)	0.83
**Treatment Line**				
1	1.0 (reference)		1.0 (reference)	
≥2	1.7 (1.2–2.1)	<0.001	1.3 (1.0–1.8)	0.040
**ECOG PS**				
<2	1.0 (reference)		1.0 (reference)	
≥2	2.3 (1.8–3.0)	<0.001	1.7 (1.2–2.2)	<0.001

CI: confidence interval, ECOG PS: Eastern Cooperative Oncology Group performance status HR: hazard ratio, LIPI: lung immune prognostic index, OS: overall survival, PFS: progression-free survival.

**Table 3:** Relationship between Lung Immune Prognostic Index (LIPI) group and objective response rate (ORR) in the pooled cohort.

ORR		LIPI Group (*n* (%))			*p* Value
		Good (0)	Intermediate (1)	Poor (2)	
**Best Response**	*PR + CR*	90 (33.0%)	48 (21.7%)	4 (4.8%)	<0.001
	*SD + PD*	183 (67.0%)	173 (78.3%)	80 (95.2%)	

CR: complete response, LIPI: lung immune prognostic index, ORR: objective response rate, PD: progressive disease, PR: partial response, SD: stable disease.

**Table 4:** Multivariate analysis of factors associated with objective response rate (ORR) in the pooled cohort.

Parameter	ORR	
	OR (95% CI)	*p* Value
**LIPI Group**		
Good (0)	1.0 (reference)	
Intermediate (1)	1.7 (1.1–2.6)	0.018
Poor (2)	9.9 (3.4–28.5)	<0.001
**Cohort**		
NSCLC	1.0 (reference)	
RCC	1.3 (0.7–2.2)	0.38
Melanoma	0.6 (0.4–1.0)	0.044
**Age**		
<70	1.0 (reference)	
≥70	0.8 (0.5–1.2)	0.21
**Treatment Line**		
1	1.0 (reference)	
≥2	2.3 (1.5–3.6)	<0.001
**ECOG PS**		
<2	1.0 (reference)	
≥2	2.0 (1.2–3.4)	0.007

CI: confidence interval, ECOG PS: Eastern Cooperative Oncology Group performance status, LIPI: lung immune prognostic index, OR: odds ratio, ORR: objective response rate.

## Supplementary Materials

The following are available online at https://www.mdpi.com/2072-6694/11/11/1713/s1, Table S1: Baseline clinical and demographic information in the non-small cell lung cancer (NSCLC), renal cell carcinoma (RCC) and melanoma cohorts. Table S2: Overall Survival (OS) and progression-free survival (PFS) based on Lung Immune Prognostic Index (LIPI) group in the non-small cell lung cancer (NSCLC), renal cell carcinoma (RCC) and melanoma cohorts. Table S3: Multivariate analysis of factors associated with overall survival (OS) and progression-free survival (PFS) in the non-small cell lung cancer (NSCLC) cohort. Table S4: Multivariate analysis of factors associated with overall survival (OS) and progression-free survival (PFS) in the renal cell carcinoma (RCC) cohort. Table S5: Multivariate analysis of factors associated with overall survival (OS) and progression-free survival (PFS) in the melanoma cohort. Table S6: Multivariate analysis of factors associated with objective response rate (ORR) in in the non-small cell lung cancer (NSCLC), renal cell carcinoma (RCC) and melanoma cohorts.
