# Supplementary material for: The Lung Immune Prognostic Index Discriminates Survival Outcomes in Patients with Solid Tumors Treated with Immune Checkpoint Inhibitors
Source: Cancers (Basel). 2019 Nov 2;11(11):1713. doi: 10.3390/cancers11111713 (PMC6896022; doi:10.3390/cancers11111713)
Supplement: Supplementary file 1 [file cancers-11-01713-s001.pdf]

cancers

MDPI

Article

The Lung Immune Prognostic Index Discriminates Survival Outcomes in Patients with Solid Tumors Treated with Immune Checkpoint Inhibitors

Daniel E. Meyers [1,†], Igor Stukalin [1,†], Isabelle A. Vallerand [1,2], Ryan T. Lewinson [1,2,3], Aleksi Suo [1], Michelle Dean [1], Scott North [4], Aliyah Pabani [1], Tina Cheng [1], Daniel Y.C. Heng [1], D. Gwyn Bebb [1] and Don G. Morris [1,*]

Table S1. Baseline clinical and demographic information in the non-small cell lung cancer (NSCLC), renal cell carcinoma (RCC) and melanoma cohorts.

Parameter	NSCLC Cohort (*N* = 302)	RCC Cohort (*N* = 145)	Melanoma Cohort (*N* = 131)
**LIPI Group—n** (%)			
Good (0)	124 (41.1)	90 (55.9)	59 (41.3)
Intermediate (1)	124 (41.1)	46 (28.6)	51 (35.7)
Poor (2)	54 (17.8)	9 (5.6)	21 (14.7)
**ECOG PS—n** (%)			
<2	191 (63.2)	123 (84.8)	111 (84.7)
≥2	107 (35.4)	19 (13.1)	19 (14.5)
unknown	4 (1.3)	3 (2.1)	1 (0.8)
**Age at start of Treatment**			
Median	65.9	64.2	63.4
Range	32–90	36–86	20–90
<70—no. (%)	184 (60.9)	107 (73.8)	88 (67.2)
≥70—no (%)	118 (39.1)	38 (26.2)	43 (32.8)
**Treatment Line—n (%)**			
1	85 (28.1)	12 (8.3)	50 (38.2)
≥2	217 (71.9)	133 (91.7)	81 (61.8)

ECOG PS: Eastern Cooperative Oncology Group performance status, LIPI: lung immune prognostic index

.

Table S2. Overall survival (OS) and progression-free survival (PFS) based on Lung Immune Prognostic Index (LIPI) group in the non-small cell lung cancer (NSCLC), renal cell carcinoma (RCC) and melanoma cohorts.

Cohort	Median OS * (95% CI)	Median PFS * (95% CI)
**NSCLC**		
Total	8.4 (6.9–10.0)	4.3 (3.4–5.1)
LIPI Good (0)	15.7 (13.2–21.6)	9.7 (6.9–11.7)
LIPI Intermediate (1)	7.1 (5.7–9.0)	3.7 (2.5–5.0)
LIPI Poor (2)	2.1 (1.4–3.0)	1.4 (1.1–2.0)
**RCC**		
Total	12.8 (7.6–19.2)	5.8 (4.1–7.8)
LIPI Good (0)	35.1 (14.3–78.0)	7.2 (5.0–11.3)
LIPI Intermediate (1)	16.1 (9.2–30.0)	4.6 (2.8–8.1)
LIPI Poor (2)	4.6 (1.2–26.5)	2.3 (0.92–4.6)
**Melanoma**		
Total	12.8 (7.6–19.2)	3.3 (2.6–6.0)
LIPI Good (0)	32.3 (24.8–NR)	14.0 (6.0–24.8)
LIPI Intermediate (1)	7.2 (3.6–12.1)	2.6 (1.9–4.1)
LIPI Poor(2)	2.4 (0.69–3.7)	1.4 (0.70–2.5)

CI: confidence interval, LIPI: lung immune prognostic index, OS: overall survival, PFS: progression-free survival; * Values expressed in months.

Table S3. Multivariate analysis of factors associated with overall survival (OS) and progression-free survival (PFS) in the non-small cell lung cancer (NSCLC) cohort.

Parameter	OS		PFS	
	HR (95% CI)	*p* Value	HR (95% CI)	*p* Value
**LIPI Group**				
Good (0)	1.0 (reference)		1.0 (reference)	
Intermediate (1)	1.6 (1.1–2.1)	0.005	1.4 (1.0–1.8)	0.037
Poor (2)	4.1(2.8–6.0)	<0.001	3.0 (2.1–4.4)	<0.001
**ECOG PS**				
<2	1.0 (reference)		1.0 (reference)	
≥2	2.9 (2.2–3.9)	<0.001	2.3 (1.7–3.0)	<0.001
**Age**				
<70	1.0 (reference)		1.0 (reference)	
≥70	0.9 (0.7–1.2)	0.59	1.0 (0.8–1.4)	0.75
**Treatment Line**				
1	1.0 (reference)		1.0 (reference)	
≥2	1.2 (0.9–1.6)	0.24	1.4 (1.0–1.8)	0.035

CI: confidence interval, ECOG PS: Eastern Cooperative Oncology Group performance status HR: hazard ratio, LIPI: lung immune prognostic index, OS: overall survival, PFS: progression-free survival.

Table S4. Multivariate analysis of factors associated with overall survival (OS) and progression-free survival (PFS) in the renal cell carcinoma (RCC) cohort.

Parameter	OS		PFS	
	HR (95% CI)	*p* value	HR (95% CI)	*p* Value
**LIPI Group**				
Good (0)	1.0 (reference)		1.0 (reference)	
Intermediate (1)	1.4 (0.8–2.4)	0.25	1.2 (0.8–1.9)	0.33
Poor (2)	3.1 (1.4–6.8)	0.004	3.8 (1.8–7.9)	<0.001
**ECOG PS**				

<2	1.0 (reference)		1.0 (reference)	
≥2	2.6 (1.4–4.9)	0.003	2.3 (1.3–3.9)	0.002
**Age**				
<70	1.0 (reference)		1.0 (reference)	
≥70	0.9 (0.5–1.5)	0.66	0.7 (0.4-1.1)	0.17
**Treatment Line**				
1	1.0 (reference)		1.0 (reference)	
≥2	4.0 (0.9–16.8)	0.056	1.3 (0.6-2.8)	0.44

CI: confidence interval, ECOG PS: Eastern Cooperative Oncology Group performance status HR: hazard ratio, LIPI: lung immune prognostic index, OS: overall survival, PFS: progression-free survival.

Table S5. Multivariate analysis of factors associated with overall survival (OS) and progression-free survival (PFS) in the melanoma cohort.

Parameter	OS		PFS	
	**HR (95% CI)**	***p* Value**	**HR (95% CI)**	***p* Value**
**LIPI Group**				
Good (0)	1.0 (reference)		1.0 (reference)	
Intermediate (1)	3.3 (2.0–5.3)	<0.001	2.2 (1.4–3.4)	<0.001
Poor (2)	7.9 (4.1–15.2)	<0.001	5.6 (3.0–10.1)	<0.001
**ECOG PS**				
<2	1.0 (reference)		1.0 (reference)	
≥2	1.4 (0.8–2.5)	0.25	1.3 (0.7–2.2)	0.39
**Age**				
<70	1.0 (reference)		1.0 (reference)	
≥70	0.6 (0.4–1.0)	0.034	0.7 (0.4–1.0)	0.070
**Treatment Line**				
1	1.0 (reference)		1.0 (reference)	
≥2	1.5 (1.0–2.4)	0.072	1.1 (0.7–1.6)	0.72

CI: confidence interval, ECOG PS: Eastern Cooperative Oncology Group performance status HR: hazard ratio, LIPI: lung immune prognostic index, OS: overall survival, PFS: progression-free survival.

Table S6. Multivariate analysis of factors associated with objective response rate (ORR) in the non-small cell lung cancer (NSCLC), renal cell carcinoma (RCC) and melanoma cohorts.

Parameter	ORR					
	NSCLC		RCC		Melanoma	
	**OR (95% CI)**	**P Value**	**OR (95% CI)**	***p* Value**	**OR (95% CI)**	***p* Value**
**LIPI Group**						
Good (0)	1.0 (reference)		1.0 (reference)		1.0 (reference)	
Intermediate (1)	1.1 (0.59–2.0)	0.80	1.3 (0.52–3.2)	0.59	5.3 (2.1–13.5)	<0.001
Poor (2)	9.1 (2.1–40.5)	0.004	*	*	11.2 (2.2–57.6)	0.004
**ECOG PS**						
<2	1.0 (reference)		1.0 (reference)		1.0 (reference)	
≥2	2.3 (0.95–5.75)	0.064	1.2 (0.46–3.1)	0.72	4.0 (1.5–10.7)	0.006
**Age**						
<70	1.0 (reference)		1.0 (reference)		1.0 (reference)	
≥70	0.92 (0.51–1.7)	0.78	0.75 (0.30–1.9)	0.54	0.52 (0.20–1.4)	0.19
**Treatment Line**						
1	1.0 (reference)		1.0 (reference)		1.0 (reference)	
≥2	2.9 (1.6–5.2)	<0.001	1.6 (0.38–7.1)	0.66	1.9 (0.77–4.6)	0.17

CI: confidence interval, ECOG PS: Eastern Cooperative Oncology Group performance status, LIPI: lung immune prognostic index, OR: odds ratio, ORR: objective response rate; * OR was not reportable for Poor LIPI (RCC) due to insufficient sample size.
